# Attitudes, Beliefs, and Self-Reported Rates of Influenza and COVID-19 Vaccinations in the Canadian 2023–2024 National Influenza and Respiratory Viruses Survey

**DOI:** 10.3390/vaccines12111230

**Published:** 2024-10-29

**Authors:** Samir Sinha, Natalie Iciaszczyk, Bertrand Roy, Wendy Boivin

**Affiliations:** 1 Section of Geriatric Medicine, Department of Medicine, Sinai Health and University Health Network, Toronto, ON M5G 1X5, Canada; 2 Division of Geriatric Medicine, Department of Medicine, University of Toronto, Toronto, ON M5S 1C5, Canada; 3 National Institute on Ageing, Toronto Metropolitan University, Toronto, ON M5B 2K3, Canada; 4 Medical Affairs Americas, CSL Seqirus, Montreal, QC H9H 4M7, Canada

**Keywords:** influenza, COVID-19, influenza vaccines, COVID-19 vaccines, vaccination coverage, vaccine perceptions

## Abstract

**Background:** We conducted a cross-sectional, online survey of adult Canadian residents to evaluate their attitudes and beliefs about vaccination against respiratory viruses, particularly influenza and coronavirus 2019 (COVID-19). **Methodology:** Survey participants aged ≥ 18 years were randomly recruited from the Léger Opinion (LEO) consumer panel. **Results:** Out of 3002 respondents, 76% reported being “up-to-date” on all of their recommended vaccinations, 86% reported understanding why the influenza vaccine was needed annually, 79% reported believing the influenza vaccine was safe, and 83% reported understanding that vaccines, in general, were important for health. However, only 49% reported receiving the influenza vaccine in the fall of 2023, and 46% received a COVID-19 vaccine (68% of those who received one received the other). More than half of the respondents (55%) reported that they found it difficult to keep track of which vaccines were recommended for them, while 74% indicated that they valued the opinion of their healthcare provider (HCP) when deciding whether to be vaccinated against influenza, and 73% said they would not hesitate to receive multiple vaccines at the same time if their HCP recommended it. **Conclusions:** These findings highlight the ongoing need for education and outreach in Canada.

## 1. Introduction

Respiratory infections cause high rates of morbidity and mortality in Canada and worldwide [1,2,3,4]. As of November 2023, coronavirus disease 2019 (COVID-19) ranked as the third-leading cause of death in Canada, and influenza and pneumonia together ranked as the eighth [5]. In addition, 19% of Canadians have reported long-term symptoms of COVID-19, causing substantial disability and other adverse impacts on health and well-being for affected individuals [6]. Both influenza and COVID-19 are responsible for significant numbers of hospitalizations in Canada [4,7], and both conditions are also strongly associated with increased cardiovascular risk, with higher rates of myocardial infarctions and strokes in people infected with either virus [8,9,10]. Altogether, the direct and indirect costs of these respiratory infections place an extremely high burden on society [3,4,11,12,13,14].

To reduce the overall burden of respiratory illnesses, the Canadian National Advisory Committee on Immunization (NACI) recommends influenza vaccination for anyone aged ≥ 6 months without a contraindication to the vaccine and prioritizes vaccination of people at high risk of influenza-related complications (including older adults aged ≥ 65 years, pregnant persons, and those with high-risk medical conditions or indigenous ancestry) and people who may transmit influenza to those at high risk of complications [4]. NACI recommends COVID-19 vaccination for all those who are unvaccinated and a currently recommended booster dose if it has been more than 6 months since the last vaccination. The group also stresses the importance of immunization for people at high risk of severe COVID-19 illness [15].

Although NACI recommendations stress the importance and safety of influenza and COVID-19 vaccines, current vaccination rates are well below the targets recommended by the Public Health Agency of Canada (PHAC). By 2025, 80% of adults aged ≥ 65 years and persons aged 18–64 years with high-risk medical conditions should be vaccinated annually against influenza, but the latest PHAC data suggest only 73% and 44% of these groups, respectively, were vaccinated during the 2023–2024 influenza season [16,17]. In the Canadian Adult National Immunization Coverage Survey (aNICS), 20% of individuals reported reluctance to receive an influenza vaccine (primarily due to the belief that it would not prevent influenza or that it was unsafe or likely to cause adverse reactions) [18]. Furthermore, although 81% of Canadians have received at least one dose of a COVID-19 vaccine, only 4% of the population is fully vaccinated per current recommendations [19]. Concerns about the safety of COVID-19 vaccines were the primary driver of COVID-19 vaccine hesitancy, which was reported by 29% of aNICS respondents [18].

Our group previously surveyed adult residents of Canada to assess their knowledge, attitudes, and uptake patterns related to influenza and COVID-19 vaccines during the 2021–2022 and 2022–2023 influenza seasons [20,21,22]. This study investigates the association between attitudes, beliefs, and self-reported rates of vaccination against respiratory illnesses in Canada using data from the Canadian 2023/2024 National Influenza and Respiratory Viruses Survey.

## 2. Materials and Methods

### 2.1. Study Design

We conducted an observational, cross-sectional, self-reported, online survey from 2–17 April 2024 of Canadian residents aged ≥ 18 years. The survey consisted of an electronic, structured questionnaire in English and French, which was accessible via multiple online platforms.

The Veritas Independent Review Board (IRB) approved the study design on 13 March 2024, and the conduct, data collection, and analysis conformed to IRB requirements and applicable laws and regulations. All survey respondents provided informed consent, and respondent information was fully anonymized before data collection, aggregation, and analysis.

### 2.2. Participants

Adult residents of Canada aged ≥ 18 years were eligible to participate in the survey. Prospective respondents were recruited from the Léger Opinion (LEO) consumer panel; invitations to participate in the survey were sent to 30,206 LEO panel members via email or push notifications via the LEO app. The invitations included a survey link unique to each invited panel member. A random selection process was used that ensured a regionally and demographically representative survey population comprising ~3000 respondents. Potential respondents were excluded if they worked or lived with someone who worked for an advertising agency; market research firm; media outlet; a manufacturer of healthcare products (over-the-counter or prescription); a drug store or company; and/or a doctor’s office, clinic, or hospital.

The integrity of the survey population was ensured by LEO registration processes that prevent multiple entries and fraudulent panelists, as previously described [22]. Fair market value compensation was offered to respondents who completed the survey where permitted by local regulations.

### 2.3. Study Objectives

The objectives of this study were to determine beliefs, awareness, and preferences around influenza vaccination for the 2023–2024 influenza season as well as regarding vaccination for other respiratory illnesses, including COVID-19, and to understand current awareness, comfort, and behavior around vaccine co-administration.

### 2.4. Survey Instrument

The online survey questionnaire consisted of screening questions, introductory messages, and contextual updates that totaled approximately 55 questions. The questions themselves were developed based on influenza survey instruments previously published by PHAC and our group [20,21,22,23]. The survey was hosted on the Decipher Survey Platform (Forsta, Vancouver, BC, Canada) and took approximately 15 min to complete. Survey domains included demographics, influenza vaccine perceptions and experience, perception and experience of vaccine administration logistics, and perceptions and experience with vaccine co-administration. Some responses led to follow-up questions, so the total number of questions seen by respondents varied depending on their answers.

Recruitment quotas by age, sex, and region were set based on 2021 Canadian census data [24], and sampling during recruitment was adjusted to ensure that collected data were representative of the Canadian population, as previously described [22]. Data were weighted by age, region, and sex based on 2021 census data [24] to further ensure representativeness.

The survey can be accessed on a website using a computer, a smartphone, or a tablet or by using a proprietary app for mobile devices. The survey was programmed to ensure that respondents answered all questions by preventing advancement from one question to the next only when respondents clicked on both an answer and the ‘continue’ button on each screen. Respondents clicked on a ‘finish’ button at the end of the survey, and respondents who answered all questions and clicked ‘finish’ were recorded as a ‘complete’ in the system.

To avoid multiple entries, respondents who clicked on a survey were assigned a unique identifier that was linked to each respondent’s LEO panel account. The system recorded whether the respondent was excluded from the survey or completed it. The system recognized the unique identifier if a respondent attempted to take the survey again and prevented the respondent from participating multiple times.

### 2.5. Handling of Missing Data and Bias

Only completed surveys, in which respondents answered all questions, were included in the analysis. Thus, there was no need to account for missing data.

As the responses were anonymized, this study was not designed to verify respondents’ reporting on vaccination history. As such, recall bias was a potential limitation of this study.

### 2.6. Statistical Methodology

Assuming a 95% confidence level, an online survey of 3000 respondents was projected to yield a margin of error of ±1.79%, 19 times out of 20. Assuming 90% of LEO panel members would meet study entry criteria and a response rate of 15%, 30,206 LEO panel members were invited to participate in this study to meet the target sample of 3000 respondents.

The analysis was performed using the statistical software Q/SPSS (Version 29) and Microsoft Excel. Relationships within the data were primarily analyzed using cross-tabulation. Primary independent variables such as vaccination status and intent to become vaccinated were analyzed along with key demographic characteristics, including age, sex, and ethnicity. The statistical significance of cross-tabulations was tested using z-test for proportions and *t*-test for means, with a 95% confidence interval (CI). Because only descriptive statistics were reported, no formal sample size calculations were performed.

## 3. Results

### 3.1. Survey Population

Out of 30,206 LEO panel members invited to participate in the survey, 3002 Canadian residents qualified and completed the questionnaire in full (response rate: 13%). The geographic and racial distribution was generally reflective of the Canadian population (Table 1). Over 80% of the respondents had at least some college education; 42% held a bachelor’s or higher degree from a university.

### 3.2. Vaccine Uptake

A total of 1464 (49%) reported receiving an influenza vaccine in the fall of 2023, whereas 2272 (76%) said they had received an influenza vaccine in a previous year. Receipt of a COVID-19 vaccine in 2023 was reported by 1377 (46%) respondents. Among participants aged 65 years and older, 548 (78%) received an influenza vaccine, and 520 (73%) received a COVID-19 vaccine in Fall 2023.

Respondents were more likely to report receiving the influenza vaccine if they were of age 65 years and older, had also received a COVID-19 vaccination, agreed that receipt of more than one vaccine with the influenza vaccine (i.e., co-administration) was safe and effective, and had insurance coverage (Table S1). Respondents were more likely to report receiving the COVID-19 vaccine if they were of age ≥ 65 years, had also received an influenza vaccine, and agreed that co-administration of vaccines was safe and effective (Table S2).

**Figure 1:**
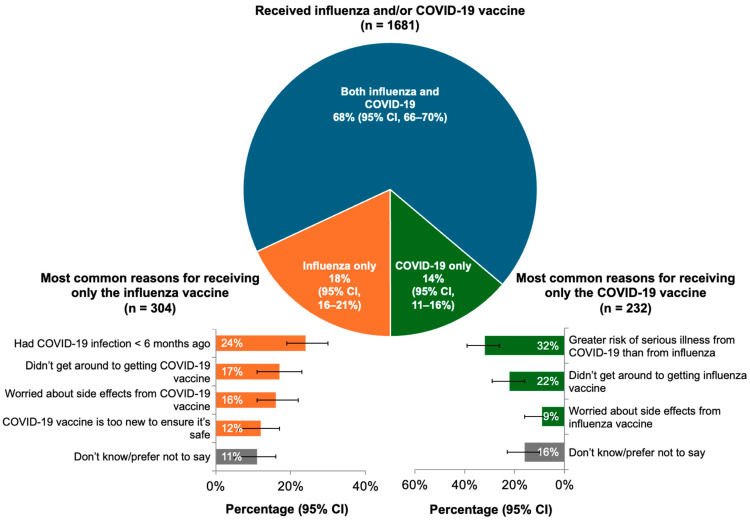
Proportions of respondents who received the influenza, COVID-19, or both vaccines and reasons given for receiving only one or the other vaccine. Error bars represent 95% confidence intervals (CI).

Among the 1145 respondents who received both an influenza and a COVID-19 vaccine in Fall 2023, 886 (77%) reported that the two vaccines were equally important. Of the 304 respondents who received the influenza vaccine only, approximately 1 in 4 were concerned about the side effects (16% [95% CI, 11–22%]) or safety (12% [95% CI, 6–17%]) of the COVID-19 vaccine (Figure 1). In contrast, the most common reason for receiving the COVID-19 vaccine but not the influenza vaccine was the perception that COVID-19 posed a greater risk of serious illness than influenza (Figure 1).

Two-thirds (65% [95% CI, 63–68%]) of the respondents reported receiving an influenza vaccine in Fall 2023 because they wanted to avoid influenza infection or illness, and half (51% [95% CI, 49–54%]) reported that they wanted to protect the health of others. More than half (55% [95% CI, 52–57%]) also reported that they receive an annual influenza vaccine out of habit (Figure S1). When asked for the primary reason for not receiving the influenza vaccine (respondents were limited to one reason), respondents most often reported reasons related to apathy about influenza vaccination (no specific reason, not getting around to it), perceptions of not needing the vaccine due to good health, and doubts about vaccine efficacy or safety (Figure S1).

### 3.3. Influenza Vaccine Attitudes and Knowledge

**Figure 2:**
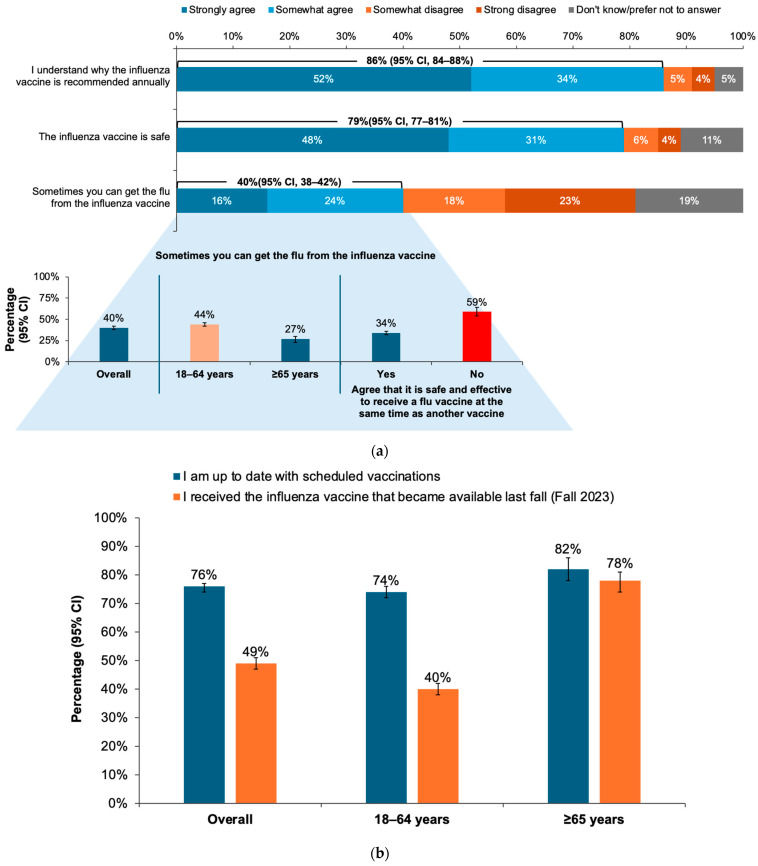
(**a**) Proportions of responses to the question, “B11. Please indicate whether you agree or disagree with each of the following statements about flu vaccination” with group agreeing that influenza vaccines cause influenza illness broken down by age and agreement/disagreement that vaccine co-administration is safe and effective. (**b**) Proportions of respondents reporting that they were up to date with scheduled vaccinations and that they received the influenza vaccine in the 2023–2024 season. Error bars represent 95% confidence intervals (CI).

Most respondents reported that they understood why the influenza vaccine is annually recommended (86% [95% CI, 84–88%]) and that they believed the influenza vaccine was safe (79% [95% CI, 77–81%]), but 40% (95% CI, 38–42%) also reported that the influenza vaccine can cause influenza illness. This belief was reported by more respondents who were younger than 65 years than those ≥65 years and was also more likely to be reported by respondents who did not agree that it was safe and effective to receive the influenza vaccine with another vaccine (Figure 2a).

A majority of respondents reported that they were up to date on all of their recommended vaccinations, yet less than half of the overall population reported having received an influenza vaccine in the 2023–2024 season. This result was driven by findings in persons younger than 65 years, of whom only 40% (95% CI, 38–42%) reported receiving the influenza vaccine, while 74% (95% CI, 72–76%) reported that their vaccinations were up to date (Figure 2b).

**Figure 3:**
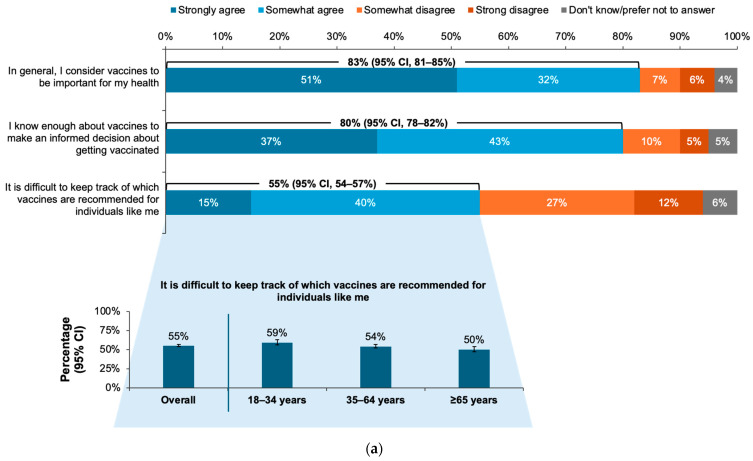

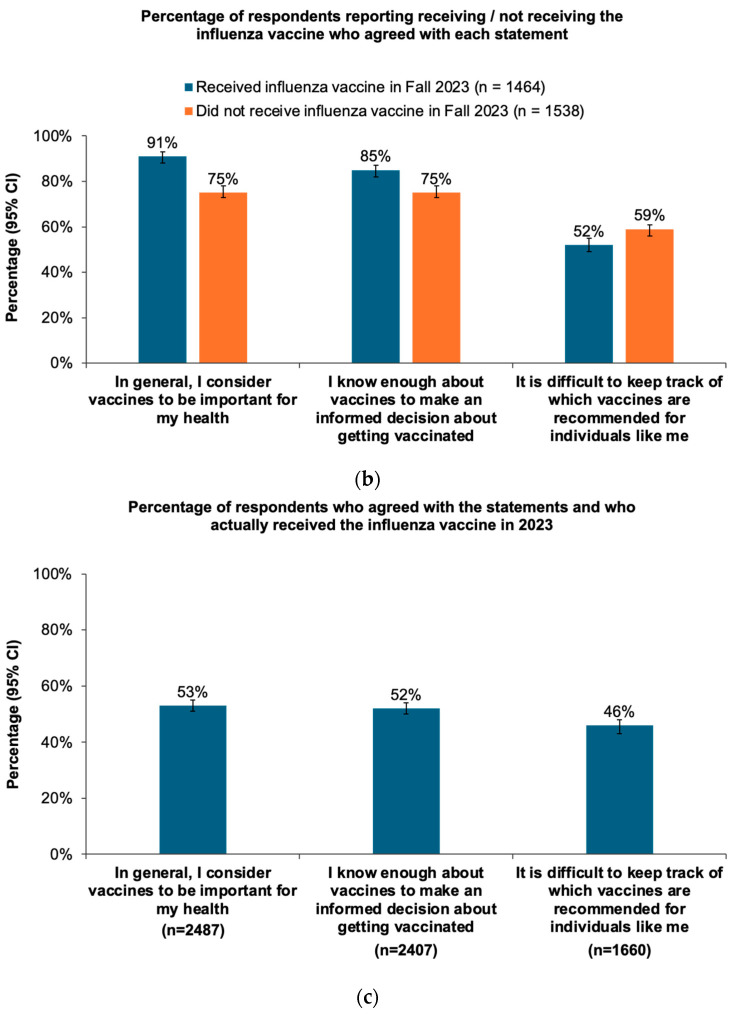
(**a**) Proportions of responses to the question, “A5. Please indicate the extent to which you agree or disagree with the following statements about vaccines in general, reporting agreement or disagreement”, with age group breakdown of respondents agreeing that keeping track of vaccine recommendations is difficult. (**b**) Percentage of respondents who agreed with the statements among those who did and did not receive an influenza vaccine in Fall 2023. (**c**) Rates of influenza vaccination in Fall 2023 among the overall group of respondents who agreed with each statement. Error bars represent 95% confidence intervals (CI).

Most respondents reported that they considered vaccines to be important for health (83% [95% CI, 81–85%]), and they were knowledgeable enough about vaccines to make informed decisions about vaccination (80% [95% CI, 78–82%]). However, 55% (95% CI, 54–57%) reported finding it difficult to keep track of their recommended vaccinations. Significantly more respondents aged 18–34 than those ≥65 years reported this difficulty (59% [95% CI, 56–63%] vs. 50% [95% CI, 47–54%], respectively; Figure 3a). Agreement with each of the three statements remained largely consistent across regions, income levels, and age groups (Table S3). The rates of agreement with the statements significantly varied with 2023 influenza vaccination status (Figure 3b). Approximately half of respondents who agreed that vaccines were important for health and that they knew enough to make informed decisions about vaccines received an influenza vaccine in Fall 2023; slightly less than half of those who found it difficult to keep track of vaccine recommendations received the Fall 2023 influenza vaccine (Figure 3c).

### 3.4. Vaccine Co-Administration Uptake and Attitudes

Among the 1,464 respondents reporting receiving the influenza vaccine in Fall 2023, 815 (55%) indicated that they received another vaccine at the same time. Among these respondents, 94% reported receiving a COVID-19 vaccine, 8% a pneumonia vaccine, and 5% a shingles vaccine (Table S4). Reasons for choosing vaccine co-administration included the following: convenience of not having to book multiple vaccination appointments (63% [95% CI, 60–67%]), they were offered additional vaccines when receiving the first vaccine (44% [95% CI, 41–48%]), they chose it to avoid missing a vaccine or booster dose (28% [95% CI, 24–31%]), and a healthcare professional (HCP) recommended vaccine co-administration (25% [95% CI, 22–29%]) (Figure S2). Of those not receiving another vaccine (n = 597), 42% (95% CI, 38–46%) reported that they were up to date on their other vaccines, 22% (95% CI, 18–26%) reported being offered only the influenza vaccine, 10% (95% CI, 6–14%) reported that they did not know vaccines can be administered at the same time, and 4% (95% CI, 0–8%) reported that their HCP had advised against co-administration (Figure S2).

**Figure 4:**
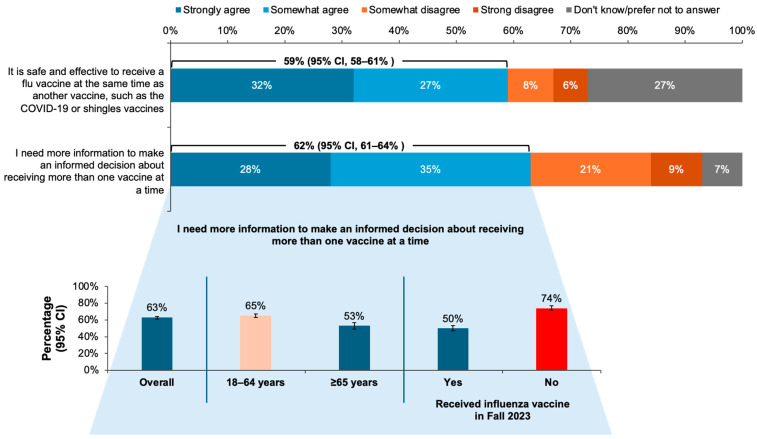
Proportions of responses to the question, “D1. It is safe and effective to receive a flu vaccine at the same time as another vaccine, such as the COVID-19 or shingles vaccines?” and “D4. Please indicate whether you agree or disagree with each of the following statements about vaccine co-administration”, with breakdown by age and 2023 influenza vaccination status for the statement about needing more information to make an informed decision. Error bars represent 95% confidence intervals (CI).

Out of the entire survey population, 59% (95% CI, 58–61%) agreed that co-administration of the influenza vaccine with another vaccine was safe and effective, although 62% (95% CI, 61–64%) reported the need for more information to make informed decisions about co-administration. Significantly more younger respondents than those aged ≥ 65 years reported the need for more information, and 74% (95% CI, 72–77%) of those who had not received an influenza vaccine in 2023 reported that they needed more information on vaccine co-administration, compared with 50% (95% CI, 47%–53%) of those who received the Fall 2023 influenza vaccine (Figure 4).

### 3.5. Trust in Healthcare Provider Advice

**Figure 5:**
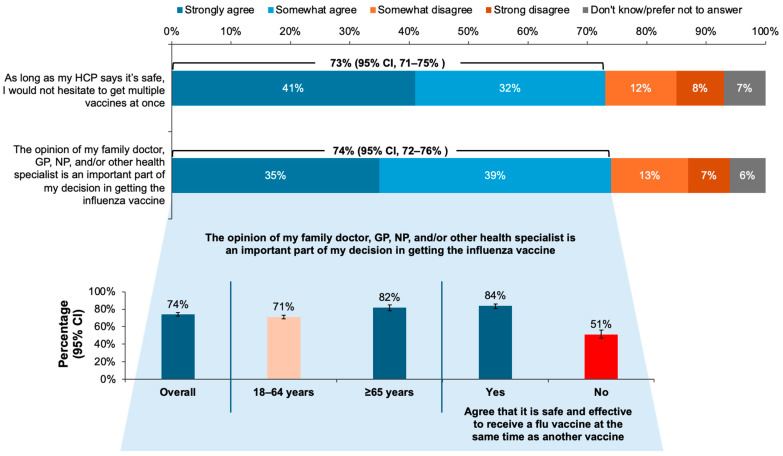
Proportions of responses to the question, “B11. Please indicate whether you agree or disagree with each of the following statements about flu vaccination” and “D4. Please indicate whether you agree or disagree with each of the following statements about vaccine co-administration”, with breakdown by age, agreement on co-administration efficacy, and safety for the statement about the importance of healthcare professionals’ (HCPs’) opinions. GP, general practitioner; NP, nurse practitioner. Error bars represent 95% confidence intervals (CI).

Three out of every four respondents (74% [95% CI, 72–76%]) reported that the opinion of a healthcare professional (HCP) was an important part of their decision to receive the influenza vaccine, and 73% (95% CI, 71–75%) reported that they would not hesitate to receive multiple vaccines at once if their HCP told them it was safe and effective. Fewer younger respondents than those aged ≥ 65 years reported this level of trust in the HCP, and more respondents who agreed that co-administration of the influenza vaccine with another vaccine was safe and effective (84% [95% CI, 81–86%]) than those who did not agree (51% [95% CI, 47–56%]) reported trusting their HCP’s opinion on vaccines (Figure 5).

### 3.6. Regional Variation

Overall, results in the provinces reflected the nationwide findings, although vaccination rates in Fall 2023 varied widely. Rates of being vaccinated against influenza at any time varied from 67% in Quebec to 80% in Alberta and British Columbia (77–78% for the other provinces; Table S1), whereas in Fall 2023, less than half of respondents from Quebec (41%) and Alberta (45%) received the influenza vaccine compared with ≥50% in the other provinces (up to 56% in British Columbia and Manitoba/Saskatchewan; Table S1). The rate of COVID-19 vaccination in Fall 2023 was lowest in Alberta (41%) and Quebec (42%), but British Columbia was the only province from which a majority of respondents reported receipt of a COVID-19 vaccine (55%) (Table S2).

There was little variation across regions in proportions of respondents agreeing that vaccines were important for health (from 80% in Alberta to 85% in the Atlantic region; Table S3). Alberta residents were the most likely (85%) and Quebec residents the least likely (76%) to report knowing enough about vaccines to make informed decisions about being vaccinated. However, fewer residents from Quebec compared with the other provinces reported difficulty keeping track of vaccine recommendations (Quebec, 45%; other provinces, 53–66%; Table S3).

Fewer Quebec residents reported valuing their HCP’s opinion when making decisions about the influenza vaccines (64% vs. 74–78% in other provinces; Table S5). Across regions, trust in HCPs’ opinion on the safety of co-administration was generally consistent with the overall result of 73% (regional rates, 71–77%; Table S5).

## 4. Discussion

In this survey, 49% of adult Canadian residents reported receiving the influenza vaccine in Fall 2023, and 46% received a COVID-19 vaccine. Influenza vaccination rates were higher among older adults; 78% of those aged ≥ 65 years received an influenza vaccine in Fall 2023 compared with only 40% of those younger than 65 years. Overall attitudes about the influenza and other vaccines were positive, with 79% of respondents agreeing that influenza vaccines were safe, 83% reporting that vaccines were important for health, 77% of those who received both an influenza and COVID-19 vaccine reporting that they were equally important, and 59% of the entire survey population reporting that co-administration of the influenza vaccine with other vaccines was safe and effective. Three quarters of the respondents reported high levels of trust in HCP opinions and recommendations regarding vaccines.

The data revealed some contrasts between the respondent’s self-reported knowledge and their attitudes and behavior regarding vaccination. Large majorities of the respondents reported understanding why the influenza vaccine was annually needed (86%) and that they knew enough about vaccines, in general, to make informed decisions (80%). However, 40% reported believing the myth that influenza vaccines can cause influenza illness. In addition, more than half of the respondents (55%) reported that they found it difficult to keep track of which vaccines were recommended for them. Contrary to self-reported vaccination rates (≤49%), 76% of the respondents reported being up to date on all scheduled vaccinations. Furthermore, only 53% of the respondents who agreed that vaccines were important for health and 52% of those reporting sufficient knowledge to make informed decisions about vaccination received an influenza vaccine in Fall 2023. These findings, along with the result that less than two-thirds of the respondents believed that vaccine co-administration was safe and effective, may help explain why PHAC vaccination goals continue to be unmet [16,17,19]. Additional education to increase confidence and understanding about vaccines for influenza and other respiratory infections is needed.

The overall influenza vaccination rates changed little from a previous survey we conducted that evaluated self-reported influenza vaccine uptake, knowledge, and attitudes during the 2021–2022 and 2022–2023 influenza seasons [20,21,22]. Some, but not all, of our current findings are consistent with the results of the PHAC 2023–2024 Seasonal Influenza Vaccination Coverage Survey—in particular, a similar proportion of respondents reported the mistaken belief that they can catch the flu from an influenza vaccine (40% in the present survey vs. 43% in PHAC) [17]. Differences between our findings and PHAC may be attributed to differences in methodology (online vs. telephone), the wording of questions, and the timing of the surveys, which may have an impact on the survey population and their responses. However, there are no other national data that permit the assessment of influenza vaccination rates in Canada. Data for COVID-19 vaccination are available from the vaccine registries in provinces and territories. In contrast to the estimates from this survey that 46% of adults received a COVID-19 vaccine in the fall of 2023, data from vaccine registries provide adult coverage estimates of 21% for Canada and 17% for Ontario [19,25]. These discrepancies raise significant questions about the validity of available vaccine coverage data for adults in Canada.

In our survey, 68% of the respondents who received the influenza and/or COVID-19 vaccine in Fall 2023 received both vaccines, and of the group who received both, 77% said that they were equally important. The most commonly cited reason for not receiving a COVID-19 vaccine among those vaccinated against influenza was a recent COVID-19 infection, but 28% of influenza vaccine–only recipients expressed concerns about the safety or tolerability of COVID-19 vaccines. The plurality of those receiving only a COVID-19 vaccine indicated a greater concern about the risks of COVID-19 illness compared with influenza (32%). In the PHAC survey, 19% of those who did not receive a COVID-19 vaccine expressed safety concerns [17]. Roughly similar proportions of those receiving only the influenza or the COVID-19 vaccine reported simply “not getting around” to receiving the other vaccine.

A key difference between the present survey and the PHAC survey was that 71% of the PHAC respondents vaccinated against influenza received a COVID-19 vaccine at the same time, while only 55% of the respondents in our survey received another vaccine (94% of which were COVID-19 vaccines) at the same time. While 42% of those who received only the influenza vaccine during their appointment indicated they were up to date on all other recommended vaccines, 22% reported that they were not offered any other vaccines during the visit, and 10% were unaware that vaccines can be administered together. These findings highlight the important role played by HCPs in fostering vaccine uptake. Our survey demonstrated a high regard and trust in HCPs’ opinions on vaccines, consistent with previous studies. Simply having an HCP doubled the likelihood of influenza vaccination among people with cardiovascular disease who participated in the Canadian Community Health Survey (CCHS) [26]. When HCPs presumptively engage with their patients about vaccines, vaccine uptake also increases. Reminding patients of the need for scheduled vaccines, including influenza and COVID-19, and taking advantage of opportunities to administer those vaccines, has been shown to positively influence rates of vaccination [27]. The Canadian Influenza Immunization Awareness Campaign offers educational resources on influenza that can help HCPs address vaccination topics with their patients, with a particular focus on those at risk of influenza complications [28].

Our findings present some contrasts and similarities with other recent surveys conducted elsewhere in the world. Similar to our results, a 2020–2021 season survey in China found that more individuals planned to receive the influenza vaccine (57%) than actually did (22%), and that there was a gap between awareness of the influenza vaccine’s existence and understanding of its function (i.e., to prevent influenza vs. preventing colds or other illness) [29]. In a survey of US military personnel and their family members conducted during the 2019–2020 season, 75% of the respondents reported believing the influenza vaccine was safe (compared with 79% of our survey), but only 26% of the US respondents reported that their HCP influenced their vaccination decision (vs. 74% in our survey) [30]. In our survey, 78% of persons aged ≥ 65 years (and thus considered at high risk of influenza complications) reported receiving the influenza vaccine, but vaccine hesitancy was high in a Greek population of high-risk individuals surveyed in the fall of 2022; only 39% of this cohort reported a firm intention to be vaccinated against influenza [31].

The data collected in this survey are limited to the self-reported information of the anonymized respondents, which may be subject to recall bias and which cannot be verified. The cross-sectional nature of this study prevents any conclusions about cause-and-effect relationships. In addition, the survey is Internet-based, and compared with the population of Canada, participants are more highly educated and more likely to be born in Canada. The low response rate of other demographic groups may have resulted in biases.

Strengths of this survey include the utilization of the LEO panel, which consists of 500,000 active members who are representative of the Canadian population. Recruitment was mostly based on random selection using diversified recruitment methods, including traditional and mobile telephone methodologies. The authors’ combined expertise in questionnaire development and survey analysis, which has been refined over several influenza seasons, is another strength.

Overall, this research underscores the need for further education and outreach in Canada. Given that more than 50% of the respondents found it difficult to keep track of which vaccines were recommended for them, and that HCP opinion was valued by roughly three quarters of the participants, a focus on increased education by HCPs can help bridge the gap in patient education. Our findings also highlight some gaps that can be explored in future research. Subsequent surveys on influenza vaccine uptake using the current study’s (or an alternative) question design may be used to further investigate self-reported influenza vaccine uptake and explore differences from the PHAC and aNICS surveys. An important effort would be to document shifts in vaccine attitudes and perceptions over periods longer than a single influenza season. A deeper exploration of the regional differences uncovered in our survey can assist in the design of targeted public health efforts in communities with low respiratory vaccine uptake and/or trust in HCPs and health authorities. In addition, investigating cultural differences in attitudes and perceptions of the efficacy and safety of respiratory vaccines, and the role of HCPs’ opinions within different populations, can also help address gaps in the awareness and understanding of respiratory vaccines and their importance for public health.

## 5. Conclusions

A majority of Canadian respondents to this survey agreed that influenza and COVID-19 vaccines are safe and effective and help prevent serious illness, and most respondents believed that they were up to date with all of their recommended vaccines. However, less than half of the respondents had received a seasonal influenza vaccine or the recommended COVID-19 booster in Fall 2023. Given our findings about the opinion of HCPs being an important part of decision-making about vaccination, HCPs have an important opportunity to help increase vaccination rates by communicating and reinforcing the benefits of annual influenza vaccination and by discussing the safety and benefits of co-administration of influenza with other vaccines with their patients.

## Figures and Tables

**Table 1:** Demographic characteristics of the survey population.

Category	Characteristic	n (%)	
		Weighted	Unweighted
Sex	Men	1441 (48)	1453 (48)
	Women	1538 (51)	1527 (51)
	Other, nonbinary, or not specified	23 (1)	22 (1)
Age	18–34 years	800 (27)	741 (25)
	35–49 years	727 (24)	747 (25)
	50–64 years	765 (25)	794 (26)
	65–79 years	537 (18)	605 (20)
	≥80 years	172 (6)	115 (4)
Race and ethnicity	White	2413 (80)	2420 (81)
	Asian	330 (11)	324 (11)
	Indigenous ^a^	100 (3)	100 (3)
	Black	93 (3)	93 (3)
	Latin American	44 (1)	43 (1)
	Other	72 (2)	72 (2)
	Prefer not to say	45 (1)	45 (1)
Region	Alberta	334 (11)	361 (12)
	Atlantic region (New Brunswick, Newfoundland, Nova Scotia, Prince Edward Island)	202 (7)	180 (6)
	British Columbia	418 (14)	400 (13)
	Manitoba, Saskatchewan	193 (6)	200 (7)
	Ontario	1162 (39)	1161 (39)
	Quebec	693 (23)	700 (23)
Born in Canada	Yes	2436 (81)	2442 (81)
	No	553 (18)	547 (18)
	Prefer not to say	14 (<1)	13 (<1)
Community size	Rural (population < 50,000)	778 (26)	782 (26)
	Small town (population 50,000–250,000)	741 (25)	736 (25)
	Large city (population 250,000–1,000,000)	786 (26)	785 (26)
	Urban (population ≥ 1,000,000)	652 (22)	655 (22)
	Not sure/Prefer not to say	45 (1)	45 (1)
Education	High school or less	542 (18)	541 (18)
	Some college or university	407 (14)	410 (14)
	College graduate or CEGEP	773 (26)	772 (26)
	Bachelor’s degree	850 (28)	847 (28)
	Master’s degree or PhD	419 (14)	421 (14)
	Prefer not to say	11 (<1)	11 (<1)
Household income	<CAN 20,000	210 (7)	205 (7)
	CAN 20,000–39,999	405 (13)	403 (13)
	CAN 40,000–69,999	609 (20)	608 (20)
	CAN 70,000–99,999	579 (19)	580 (19)
	CAN 100,000–119,999	343 (11)	340 (11)
	≥CAN 120,000	587 (20)	588 (20)
	Prefer not to answer	270 (9)	278 (9)
Insurance coverage	Public/provincial	1494 (50)	1502 (50)
	Private	1177 (39)	1173 (39)
	None	210 (7)	208 (7)
	Don’t know/prefer not to say	121 (4)	119 (4)

^a^ Includes First Nations, Métis, Inuk. CEGEP, Collège d’Enseignement Général et Professionnel (general and vocational college in Quebec).

## Data Availability

Data available from the authors upon request.

## Supplementary Materials

The following supporting information can be downloaded at: https://www.mdpi.com/article/10.3390/vaccines12111230/s1, Survey Instrument; Table S1. Percentage of respondents who received the influenza vaccine in Fall 2023 or at any time previously; Table S2. Percentage of respondents who received the COVID-19 vaccine in Fall 2023; Figure S1. Most common answers to questions about why respondents did or did not receive the influenza vaccine; Table S3. Percentage of respondents agreeing with statements about recommended vaccination; Table S4. Vaccines received with influenza vaccine, as reported by respondents who received ≥ 1 vaccine in addition to their influenza vaccine; Figure S2. Most common answers to questions about reasons why respondents did or did not choose vaccine co-administration; Table S5. Percentage of respondents agreeing with the statements in the left-most column of the table.
