# Supplementary material for: Attitudes, Beliefs, and Self-Reported Rates of Influenza and COVID-19 Vaccinations in the Canadian 2023–2024 National Influenza and Respiratory Viruses Survey
Source: Vaccines (Basel). 2024 Oct 29;12(11):1230. doi: 10.3390/vaccines12111230 (PMC11598468; doi:10.3390/vaccines12111230)
Supplement: Supplementary file 1 [file vaccines-12-01230-s001.zip › vaccines-3257162-supplementary.pdf]

SUPPLEMENTARY MATERIALS

Survey Instrument

MAIN QUESTIONNAIRE

SECTION A: VACCINATION IN GENERAL

A1. As far as you know, are you up to date on your recommended vaccines?

Yes	1
No	2
Don't know/Not sure	98
Prefer not to say	99

A2. Have you received a dose of the latest COVID-19 vaccine that became available last fall (Fall 2023)?

Yes	1
No, I had COVID-19 infection in the past 6 months, before I could get last fall's COVID-19 vaccine	2
No, I had my last COVID-19 vaccine was before October 2023	3
No, I did not get it for another reason	4
Don't know/Not sure	98
Prefer not to say	99

A3. Are you aware there is a vaccine for the respiratory syncytial virus (RSV)?

Yes	1
No	2

PN: SHOW THE BELOW ON A SEPARATE SCREEN AFTER A3
Respiratory syncytial virus (also known as RSV) is a respiratory virus that usually causes mild, cold-like symptoms. However, among very young children and older adults RSV can be serious and require hospitalization. A vaccine is available in Canada to protect older adults from severe RSV.

PN: ASK IF 60+ YEARS AT S1
A4a. Have you been vaccinated against RSV?

Yes	1
No	2
Prefer not to say	99

PN: ASK IF YES (CODE 1) AT A4a

A4b. Did you pay for the RSV vaccine you received?

Yes	1
No	2
Don’t know/Not sure	98
Prefer not to say	99

A5. Please indicate the extent to which you agree or disagree with the following statements about vaccines in general.

PN: RANDOMIZE

		Strongly disagree	Somewhat disagree	Somewhat agree	Strongly agree	Don’t know/Not sure	Prefer not to say
1	In general, I consider vaccines to be important for my health	1	2	3	4	98	99
2	I know enough about vaccines to make an informed decision about getting vaccinated	1	2	3	4	98	99
4	It is difficult to keep track of which vaccines are recommended for individuals like me	1	2	3	4	98	99

<u>SECTION B: ADULT — FLU VACCINATION</u>

We would now like to ask you some questions about the flu vaccine, and your vaccination status.

B1. Have you ever received the flu vaccine?

Yes	1
No	2

PN: ASK IF CODE 1 SELECTED AT B1

B2. Did you receive the latest annual flu vaccine that became available last fall (Fall 2023)?

Yes	1
No	2

PN: ASK IF CODE 1 SELECTED AT B1

B3. Where did you receive your last flu shot? *Select only one.*

Place of employment	1
Pharmacy	2
Physician’s office or medical clinic	3
Hospital	4
Community-based public health clinic, such as in a shopping mall or library / CLSC **[ONLY FOR QC]**	5
Public Health Department	6
Other (specify)	96
Can’t remember	98
Prefer not to say	99

PN: ASK IF CODE 1 SELECTED AT B1

B4. Where would you prefer to get a flu shot? *Select as many as 3.*

Place of employment	1
Pharmacy	2
Physician’s office or medical clinic	3
Hospital	4
Community-based public health clinic, such as in a shopping mall or library / CLSC **[ONLY FOR QC]**	5
Public Health Department	6
Other (specify)	96
Don’t know/Not sure	98
Prefer not to say	99

PN: ASK IF CODE 1 SELECTED AT B2

B5. What are the reasons you decided to receive the latest annual flu vaccine that became available last fall (Fall 2023)? *Select all that apply.*

PN: RANDOMIZE

I want to prevent infection / I do not want to get sick	2
I am at risk because of my health condition	3
I am at risk because of my age	4
I receive it every year / It’s just something I’ve always done	5
To protect the health of others	6
It’s recommended by my health care provider	7
It was encouraged by family members, colleagues or friends	8
It’s free	9
Other (specify): ____________________	96

Don’t know/Not sure	98
Prefer not to say	99

PN: ASK IF CODE 2 SELECTED AT B2

B6. What was the *most important* reason why you did not receive the latest flu vaccine that became available last fall (Fall 2023)? *Select only one.*

PN: RANDOMIZE

I don’t believe in vaccines	1
Flu vaccines don’t work	2
I am healthy, and/or never get the flu	3
Getting the flu doesn’t make me that sick	4
I did not get around to it	5
I have concerns about the flu vaccine, and/or its side effects	6
I got the flu before I had the opportunity to get the flu shot	9
It was too expensive (cost of the vaccine)	10
I was not able to get an appointment	11
No specific reason, I just didn’t get it	12
The flu vaccine was out of stock when I tried to get it	13
Other (specify): _____________	96
Don’t know/Not sure	98
Prefer not to say	99

PN: ASK IF CODE 1 AT A2 (COVID-19 VACCINE) <u>AND</u> CODE 1 AT B2 (FLU VACCINE)

B7. Which vaccine was most important for you to get this past fall (Fall 2023)?

Influenza	1
COVID-19	2
Both are equally important	3

PN: ASK IF CODE 2 AT A2 (NO COVID-19 VACCINE) <u>AND</u> CODE 1 AT B2 (FLU VACCINE)

B8a. What is the most important reason you chose to get your influenza vaccine but not your COVID-19 vaccine? *Select one only.*

PN: RANDOMIZE

I had COVID-19 infection less than 6 months ago, so I’m not due for a COVID-19 vaccine	1
I am at a greater risk of serious illness from influenza than COVID-19	2
The COVID-19 vaccine is more likely than the influenza vaccine to give me side effects	3
COVID-19 vaccines are still too new to be sure they are safe	4

I'm worried about side effects from the COVID-19 vaccine	5
I didn't get around to getting my COVID-19 vaccine	7
Other (specify): _______	96
Don't know/Not sure	98
Prefer not to say	99

PN: ASK IF CODE 1 AT A2 (COVID-19 VACCINE) AND CODE 2 AT B2 (NO FLU VACCINE)

B8c. What is the most important reason you chose to get your COVID-19 vaccine but not your influenza vaccine? *Select one only*.

PN: RANDOMIZE

I am at greater risk of serious illness from COVID-19 than influenza	1
The influenza vaccine is more likely than the COVID-19 vaccine to give me side effects	2
I'm worried about side effects from the influenza vaccine	3
I didn't get around to getting my influenza vaccine	5
Other (specify): _______	96
Don't know/Not sure	98
Prefer not to say	99

B9. How worried, if at all, were you about getting the flu during this past flu season (Fall/Winter 2023/24)?

Not at all worried 1	2	3	4	Very worried 5	Don't know/ Not sure 98	Prefer not to say 99

B10. Do you intend to receive the flu vaccine this coming fall (October-December of 2024)?

Yes, definitely	1
Yes, probably	2
Probably not	3
Definitely not	4
Don't know/Not sure	98
Prefer not to say	99

B11. Please indicate whether you agree or disagree with each of the following statements about flu vaccination.

PN: RANDOMIZE

		Strongly disagree	Somewhat disagree	Somewhat agree	Strongly agree	Don't know/Not sure	Prefer not to say
2	Sometimes, you can get the flu from the flu vaccine	1	2	3	4	98	99
3	The opinion of my family doctor, general practitioner, nurse practitioner and/or other health specialists is an important part of my decision when it comes to getting the flu vaccine	1	2	3	4	98	99
4	The flu vaccine is safe	1	2	3	4	98	99
5	I understand why the flu vaccine is recommended annually	1	2	3	4	98	99

SECTION C: ENHANCED FLU VACCINE

We would now like to understand your awareness, perceptions, and experiences with different types of flu vaccines.

C1. Did you know that each year more than one type of flu vaccine is approved for use in Canada?

Yes	1
No	2

C2. Did you know that there are different influenza vaccines made for adults less than 65 years of age and for people 65 years of age and older?

Yes, definitely	1
Yes, vaguely	2
No	3

PN: SHOW THE BELOW ON A SEPARATE SCREEN AFTER C2

There are, in fact, different influenza vaccines made for younger adults and for people 65 years of age and older. Enhanced influenza vaccines are designed to give older adults better protection against the flu.

C3. Before today, did you know that there is more than one type of enhanced influenza vaccine made specifically for people 65 years of age and older?

Yes, definitely	1
Yes, vaguely	2
No	3

PN: SHOW THE BELOW ON A SEPARATE SCREEN AFTER C4
There is, in fact, more than one type of enhanced influenza vaccine specifically formulated and approved for use in for people 65 years of age and older. A higher dose, or the addition of an adjuvant, which is an ingredient that enhances the body's immune response, can be used.

PN: ASK IF CODE 1 SELECTED AT B1 AND 65+ YEARS AT S1
C4. As far as you are aware, have you **ever** received an enhanced influenza vaccine for adults 65 years of age and older?

Yes	1
No, but I tried to get it	2
No, I have not tried to get it	3
Don't know / Not sure	99

PN: ASK IF CODE 1 SELECTED AT B2 AND 65+ YEARS AT S1
C4a. As far as you are aware, did you receive an enhanced influenza vaccine for adults 65 years of age and older that became available **last fall** (Fall 2023)?

Yes	1
No, but I tried to get it	2
No, I have not tried to get it	3
Don't know / Not sure	99

PN: ASK ALL
C5. Please indicate whether you agree with each of the following statements about enhanced influenza vaccines for adults 65 years of age and older.

		Strongly disagree	Somewhat disagree	Somewhat agree	Strongly agree	Don't know/Not sure	Prefer not to say
1	Some enhanced influenza vaccines are better than others	1	2	3	4	98	99

3	It is important that older Canadians have access to enhanced influenza vaccines	1	2	3	4	98	99
4	Enhanced influenza vaccines should be available free of charge to any older Canadian who wants one	1	2	3	4	98	99

PN: ASK IF 65+ YEARS AT S1

C6. **[IF NO COVERAGE (PROV: QUEBEC)]:** Assuming your province/territory offered enhanced influenza vaccines for adults 65 years of age and older free of cost, would it make you more likely to get vaccinated against the flu?
[IF COVERAGE (PROV: non-QUEBEC)]: Since your province/territory offers enhanced influenza vaccines for adults 65 years of age and older free of cost, are you more likely to get vaccinated against the flu?

Much more likely	1
Somewhat more likely	2
No change in likelihood	3
Somewhat less likely	4
Much less likely	5
Don't know/Not sure	98
Prefer not to say	99

ASK ALL

C7. **IF YES (CODE 1) AT C4 (GOT ENHANCED VACCINE):** How satisfied were you with your experience in the following aspects of obtaining your **most recent** enhanced influenza vaccine for adults 65 years of age and older?
IF YES (CODE 1) AT B1 AND NOT ASKED C4 OR ANSWERED CODE 2, 3, OR 99 AT C4 (DID NOT GET ENHANCED VACCINE): How satisfied were you with your experience in the following aspects of obtaining your **most recent** influenza vaccine?

PN: RANDOMIZE

		Very dissatisfied	Somewhat dissatisfied	Somewhat satisfied	Very satisfied	Don't know/Not sure	This doesn't apply to me

1	Finding information on the **[65+ YEARS:** enhanced**]** influenza vaccine	1	2	3	4	98	99
2	Finding a location that has available **[65+ YEARS:** enhanced**]** influenza vaccines (i.e., in stock)	1	2	3	4	98	99
3	Process of booking an appointment	1	2	3	4	98	99
4	Ease of getting to the location to get the **[65+ YEARS:** enhanced**]** influenza vaccine	1	2	3	4	98	99

PN: ASK IF CODE 2 AT C4a

C8. You mentioned that you tried to get an enhanced influenza vaccine that became available last fall (Fall 2023) but could not. Did the challenges to accessing the enhanced influenza vaccine prevent you from receiving it (i.e., out of stock)?

Yes	1
No	2
Don't know / Not sure	99

<u>SECTION D: CO-ADMINISTRATION OF INFLUENZA, COVID-19, AND RSV VACCINATION</u>

D1. It is safe and effective to receive a flu vaccine at the same time as another vaccine, such as the COVID-19 or shingles vaccines.

Strongly disagree	Somewhat disagree	Somewhat agree	Strongly agree	Don't know/Not sure
1	2	3	4	98

The co-administration of vaccines involves receiving multiple vaccines during the same healthcare visit. The next few questions will be around the co-administration of vaccines.

PN: ASK IF YES (CODE 1) AT B2

D2. You mentioned earlier that you got the flu vaccine that became available last fall (Fall 2023). Did you get the flu vaccine and another vaccine at the same time?

Yes	1
No	2
Don't know / Not sure	98
Prefer not to say	99

PN: ASK IF SELECTED YES (CODE 1) AT D2

D2a. Which of the following vaccines did you receive at the same time as your flu vaccine this past fall (Fall 2023)? *Select all that apply.*

COVID-19 vaccine	1
Pneumonia vaccine	2
Shingles vaccine	3
RSV vaccine	4
Other (specify)	96
Don't know / Not sure	98
Prefer not to say	99

PN: ASK IF SELECTED YES (CODE 1) AT D2

D3a. What was the reason(s) that you received another vaccine with your flu vaccine this past fall (Fall 2023)? *Select all that apply.*

PN: RANDOMIZE

I was offered another vaccine when getting my flu vaccine	2
I was offered the flu vaccine when getting another vaccine	3
A healthcare professional (such as a doctor or pharmacist) recommended it	4
It saves me time from booking multiple appointments	5
Receiving multiple vaccines at once reduces the risk that I might miss a dose or vaccine	6
Other (specify)	96
Prefer not to say	99

PN: ASK IF SELECTED NO (CODE 2) AT D2

D3b. What was the reason(s) that you did not receive another vaccine with your flu vaccine this past fall (Fall 2023)? *Select all that apply.*

PN: RANDOMIZE

I was only offered the flu vaccine	1
I did not know you can receive other vaccines with the flu vaccine	2

I wanted to receive another vaccine with my flu vaccine, but couldn't because they did not have it in stock at the time	3
I wanted to receive another vaccine with my flu vaccine, but a healthcare provider advised against it	4
I was already up-to-date on all my other vaccines, and only needed the flu vaccine	5
I am concerned that the vaccines won't work as well if they are given at the same time	6
Other (specify)	96
Prefer not to say	99

D4. Please indicate whether you agree or disagree with each of the following statements about vaccine co-administration.

PN: RANDOMIZE

		Strongly disagree	Somewhat disagree	Somewhat agree	Strongly agree	Don't know/Not sure	Prefer not to say
2	As long as my health care provider says its safe, I would not hesitate to get multiple vaccines at once	1	2	3	4	98	99
3	I need more information to make an informed decision about receiving more than one vaccine at a time	1	2	3	4	98	99

SECTION E: DEMOGRAPHICS

E1. Please provide the first half of your postal code (e.g. K1K). [Open-end]

_____ (A2A)

E2. People living in Canada come from many different cultural and racial backgrounds. The following question will help us to better understand the experiences of the communities that we serve. Do you consider yourself to be . . . *Select all that apply.*

First Nation	1
Inuit	2
Métis	3
Indigenous/Aboriginal (not included above)	4

Arab	5
Black (North American, Caribbean, African, etc.)	6
Chinese	7
Filipino	8
Japanese	9
Korean	10
Latin American	11
South Asian (East Indian, Pakistani, Sri Lankan etc.)	12
Southeast Asian (Vietnamese, Cambodian, Malaysian, Laotian, etc.)	13
West Asian (Iranian, Afghan, etc.)	14
White (North American, European, etc.)	15
Other (specify): ____________________	96
Prefer not to say	99

E3. What is the highest level of education you have completed?

Less than high school	1
High school	2
Some college or university	3
College graduate or CEGEP	4
Bachelor’s degree	5
Master’s or professional degree	6
Doctorate	7
Prefer not to say	99

E4. Which of the following best describes your current health insurance coverage?

Public / provincial coverage	1
Private insurance	2
No coverage	3
Don’t know/Not sure	98
Prefer not to say	99

E5. What is your annual household income (from all sources before taxes)?

Less than $20,000	1
$20,000-$39,999	2
$40,000-$69,999	3
$70,000-$99,999	4
$100,000-$119,999	5

$120,000 or more	6
Prefer not to say	99

E6. Which of the following location descriptions best defines where you live? *Select one only.*

Rural (population of less than 50,000)	1
Small town (population between 50,000 – 250,000)	2
Large city (population from 250,000 – 1 million)	3
Metropolitan (population of 1 million or more)	4
Don't know/Not sure	5
Prefer not to say	6

E7. Were you born in Canada?

Yes	1
No	2
Prefer not to say	99

PN: ASK IF NO AT E7

E8. In which country were you born?

Afghanistan	1
Algeria	2
Bangladesh	3
Belgium	4
China	5
Colombia	6
France	7
Germany	8
Greece	9
Guyana	10
Hong Kong	11
India	12
Iran	13
Italy	14
Jamaica	15
Korea, Republic of (South Korea)	16
Lebanon	17
Netherlands	18
Pakistan	19
Philippines	20
Poland	21
Portugal	22

Romania	23
Russia	24
Sri Lanka	25
Taiwan	26
Trinidad and Tobago	27
Turkey	28
Ukraine	29
United Kingdom	30
United States	31
Vietnam	32
Other (please specify) _____	96
Don't know/Not sure	98
Prefer not to say	99

PN: ASK IF NO AT E7

E9. In what year did you move to Canada?

Record year: XXXX

- Don't know/Not sure
- Prefer not to say

Thank you for participating in the survey. This brings us to the end of the survey.

Table S1. Percentage of respondents who received the influenza vaccine in Fall 2023 or at any time previously.

n (%)		Region						Age		Received COVID-19 vaccine that became available Fall 2023		Coadministration of influenza vaccine with another vaccine is safe and effective	
	**Overall (N=3,002)**	**AB (n=361)**	**ATL (n=180)**	**BC (n=400)**	**MB/SK (n=200)**	**ON (n=1,161)**	**QC (n=700)**	**18-64 years (n=2,282)**	**≥65 years (n=720)**	**Yes (n=1,377)**	**No (n=1,558)**	**Agree (n=1,788)**	**Disagree (n=407)**
Fall 2023[a]	1,464 (49)	166 (45)	99 (54)	227 (56)	111 (56)	568 (50)	293 (41)	916 (40)	548 (78)	1,145 (83)	304 (20)	1,153 (64)	106 (26)
Ever before[b]	2,272 (76)	289 (80)	142 (78)	323 (80)	158 (79)	891 (77)	469 (67)	1,641 (72)	631 (88)	1,267 (92)	966 (62)	1,538 (86)	227 (56)

[a]From B2. Did you receive the latest annual flu vaccine that became available last fall (Fall 2023)?

[b]From B1. Have you ever received the flu vaccine?

AB, Alberta; ATL, Atlantic region (New Brunswick, Newfoundland, Nova Scotia, Prince Edward Island); BC, British Columbia; MB/SK, Manitoba/Saskatchewan; ON, Ontario; QC, Quebec.

Table S2. Percentage of respondents who received the COVID-19 vaccine in Fall 2023.

n (%)	Overall (N=3,002)	Region						Age		Received influenza vaccine in Fall 2023		Coadministration of influenza vaccine with another vaccine is safe and effective	
		AB (n=361)	ATL (n=180)	BC (n=400)	MB/SK (n=200)	ON (n=1,161)	QC (n=700)	18-64 years (n=2,282)	≥65 years (n=720)	Yes (n=1,464)	No (n=1,538)	Agree (n=1,788)	Disagree (n=407)
Fall 2023[a]	1,377 (46)	149 (41)	84 (46)	219 (55)	93 (48)	534 (46)	298 (42)	857 (37)	520 (73)	1,145 (78)	232 (15)	1,080 (60)	109 (27)

[a]From A2. Have you received a dose of the latest COVID-19 vaccine that became available last fall (Fall 2023)?

AB, Alberta; ATL, Atlantic region (New Brunswick, Newfoundland, Nova Scotia, Prince Edward Island); BC, British Columbia; MB/SK, Manitoba/Saskatchewan; ON, Ontario; QC, Quebec.

Figure S1. Most common answers to questions about why respondents did (**a**) or did not (**b**) receive the influenza vaccine in Fall 2023. (**a**) "B5. What are the reasons you decided to receive the latest annual flu vaccine that became available last fall (Fall 2023)? Select all that apply." (**b**) "B6. What was the most important reason why you did not receive the latest flu vaccine that became available last fall (Fall 2023)? Select only one." *Entry for "Other" open answer field. Error bars represent 95% confidence intervals (CI).

a

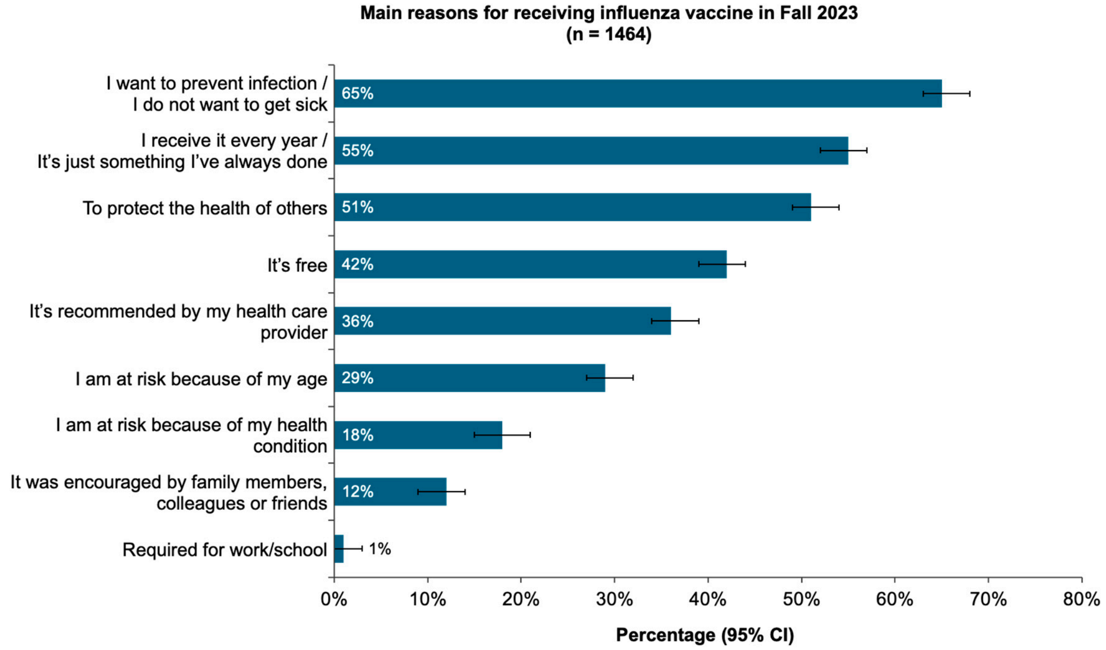

b

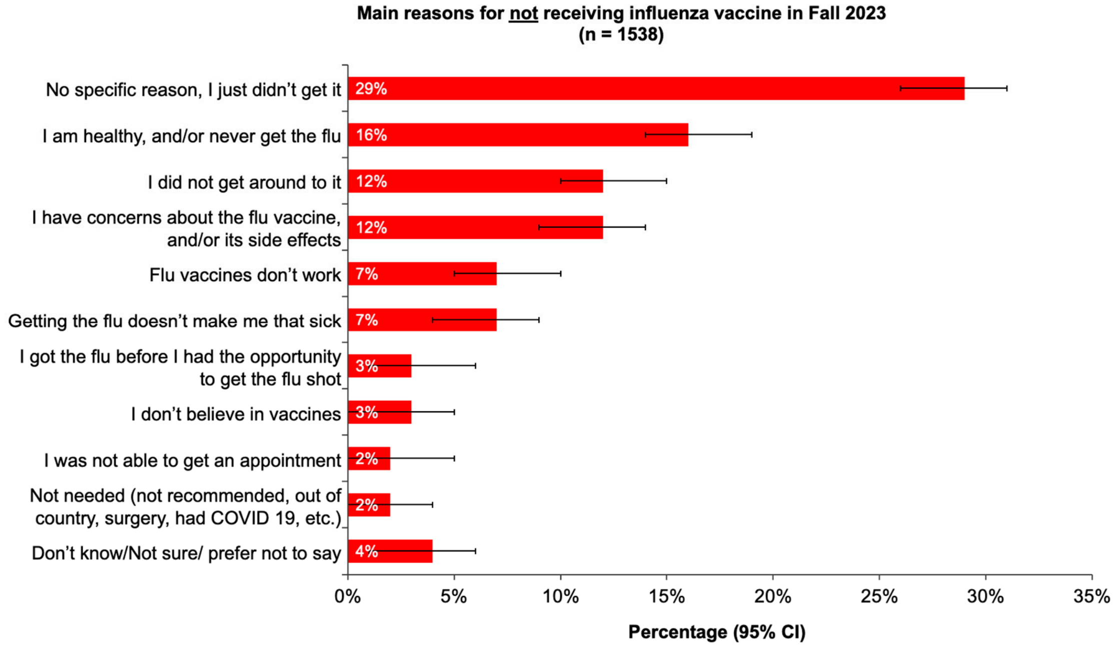

Table S3. Percentage of respondents agreeing with statements about recommended vaccination.[a]

n (%)		Region						Household income				Age	
	**Overall (N=3,002)**	**AB (n=361)**	**ATL (n=180)**	**BC (n=400)**	**MB/SK (n=200)**	**ON (n=1,161)**	**QC (n=700)**	**<$40K (n=608)**	**$40-$69K (n=608)**	**$70-$99K (n=580)**	**≥$100K (n=928)**	**18-64 years (n=2,282)**	**≥65 years (n=720)**
In general, I consider vaccines to be important for my health	2,487 (83)	292 (80)	154 (85)	327 (82)	163 (81)	969 (83)	582 (83)	478 (79)	511 (84)	492 (85)	784 (84)	1,848 (81)	639 (89)
I know enough about vaccines to make an informed decision about getting vaccinated	2,407 (80)	309 (85)	140 (78)	325 (81)	165 (82)	939 (81)	529 (76)	459 (75)	483 (79)	471 (81)	774 (83)	1,796 (79)	611 (85)
It is difficult to keep track of which vaccines are recommended for individuals like me	1,660 (55)	190 (53)	119 (66)	228 (57)	120 (59)	693 (60)	310 (45)	328 (54)	341 (56)	340 (59)	504 (54)	1,301 (57)	359 (50)

[a]From A5. Please indicate the extent to which you agree or disagree with the following statements about vaccines in general.

AB, Alberta; ATL, Atlantic region (New Brunswick, Newfoundland, Nova Scotia, Prince Edward Island); BC, British Columbia; MB/SK, Manitoba/Saskatchewan; ON, Ontario; QC, Quebec.

Table S4. Vaccines received with influenza vaccine, as reported by respondents who received ≥1 vaccine in addition to their influenza vaccine.[a]

**Vaccine type, n (%)**	**Respondents who received influenza plus ≥1 additional vaccine at the same time (n = 815)**
COVID-19	767 (94)
Pneumonia	61 (8)
Shingles	38 (5)
RSV	30 (4)
Tetanus	6 (0.6)
Other	4 (0.5)
Don't know/not sure	8 (1)
Prefer not to say	1 (0.1)

RSV, respiratory syncitial virus.
[a]From: D2a. Which of the following vaccines did you receive at the same time as your flu vaccine this past fall (Fall 2023)? Select all that apply.

Figure S2. Most common answers to questions about reasons why respondents did or did not choose vaccine coadministration. “D3a. What was the reason(s) that you received another vaccine with your flu vaccine this past fall (Fall 2023)? Select all that apply” (**a**) and “D3b. What was the reason(s) that you did not receive another vaccine with your flu vaccine this past fall (Fall 2023)? Select all that apply” (**b**). Error bars represent 95% confidence intervals (CI).

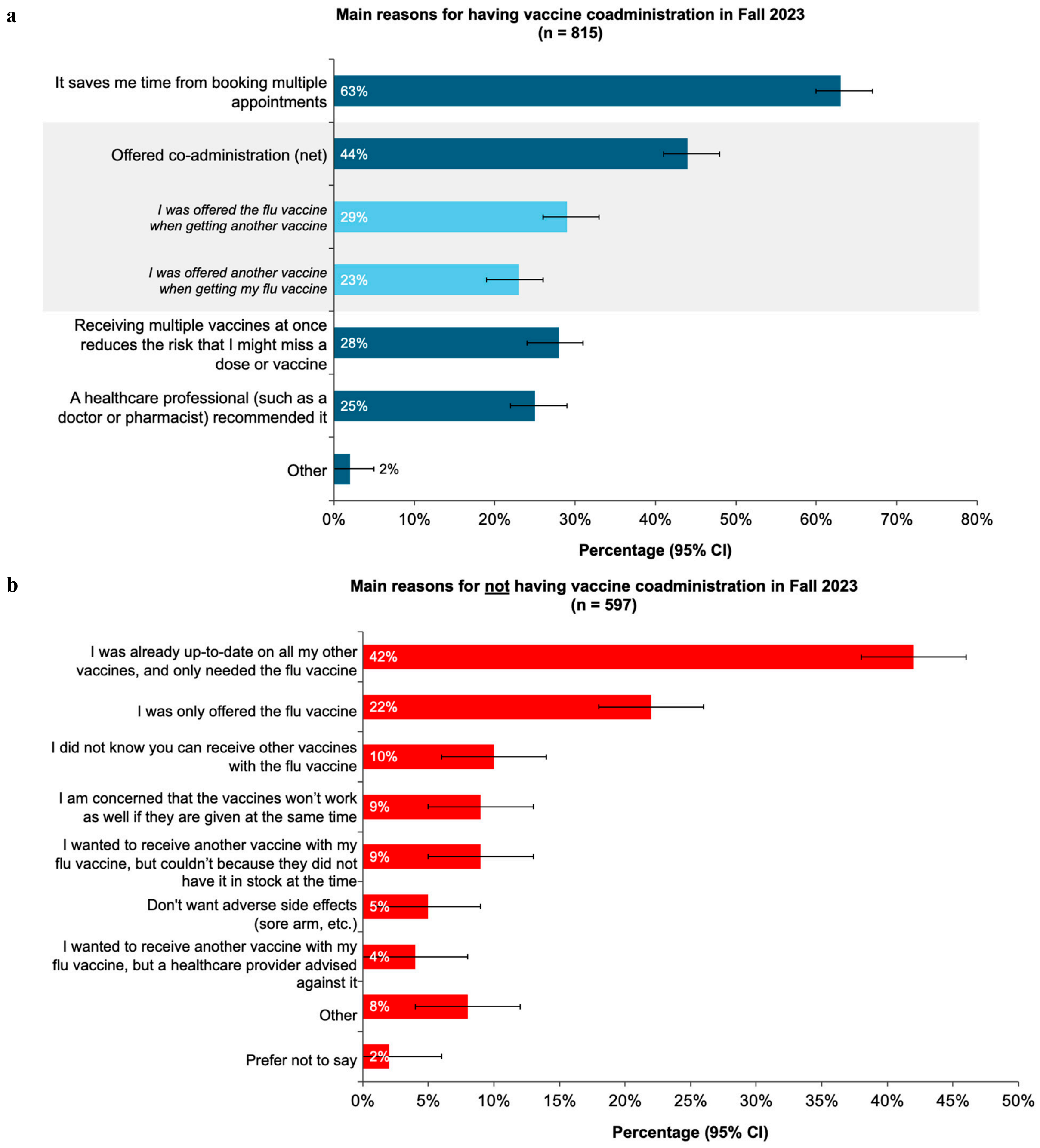

Table S5. Percentage of respondents agreeing with the statements in the left-most column of the table.[a]

		Region					
	**Overall (N=3,002)**	**AB (n=361)**	**ATL (n=180)**	**BC (n=400)**	**MB/SK (n=200)**	**ON (n=1,161)**	**QC (n=700)**
Opinion of my family Dr, GP, NP and/or other health specialists is an important part of my decision in getting the flu vax[a]	2,212 (74)	268 (74)	138 (77)	315 (78)	152 (76)	890 (77)	449 (64)
As long as my HCP says it's safe, I would not hesitate to get multiple vaccines at once[b]	2,192 (73)	256 (71)	130 (72)	297 (74)	154 (77)	850 (73)	505 (72)

[a]From B11. Please indicate whether you agree or disagree with each of the following statements about flu vaccination.
[b]From D4. Please indicate whether you agree or disagree with each of the following statements about vaccine co-administration.
AB, Alberta; ATL, Atlantic region (New Brunswick, Newfoundland, Nova Scotia, Prince Edward Island); BC, British Columbia; MB/SK, Manitoba/Saskatchewan; ON, Ontario; QC, Quebec.
